# Trichobezoar Causing Obstruction of a Percutaneous Endoscopic Gastrostomy (PEG) Feeding Tube: A Case Report

**DOI:** 10.7759/cureus.98679

**Published:** 2025-12-08

**Authors:** Ahmed Elkhalifa, Mohamed A Elfeky, Serene Khabbass, Bahaa A Qadas, Samir Abdulla

**Affiliations:** 1 General and Colorectal Surgery, The Royal Oldham Hospital, Northern Care Alliance NHS Foundation Trust, Oldham, GBR; 2 General Surgery, Faculty of Medicine, Tanta University, Tanta, EGY

**Keywords:** forigen body, gastric trichobezoar, trichobezoar and learning disability, trichobezoar in adolescents, trichobezoar in psychatric patient

## Abstract

Trichobezoar (hairball) is a rare condition that may pose a diagnostic challenge. Trichobezoar is prevalent in young females with psychiatric disorders. It is defined as a compact mass of ingested hair that accumulates within the gastrointestinal (GI) tract, most commonly in the stomach, and can sometimes extend into the intestines. Its formation occurs because hair is indigestible and tends to mat together over time, creating a dense, often obstructive mass. When a trichobezoar extends beyond the duodenum, the condition is referred to as Rapunzel syndrome. Trichobezoars are often associated with behavioral disorders such as trichotillomania (hair-pulling) and trichophagia (hair-swallowing), which may occur unconsciously or as part of impulse control disorders. This condition is particularly prevalent among adolescent females with psychiatric disorders.

A 29-year-old female with learning disabilities presented to her general practitioner with an obstructed percutaneous endoscopic gastrostomy (PEG) feeding tube and was referred for an elective gastroscopy for a presumed dislodged PEG tube. During the procedure, a foreign body resembling a kitchen scrubber was identified, causing partial obstruction of the duodenal bulb (D1) and the second part of the duodenum (D2) and resulting in ulceration in D2. The foreign body was stuck to the inner part of the PEG tube. The endoscopist successfully freed the PEG tube and replaced it with a new one; however, multiple attempts to retrieve the foreign body were unsuccessful.

A post-procedural abdominal CT scan demonstrated a presumed metallic foreign body within the stomach, measuring approximately 8 mm in length and 4 mm in thickness. The case was discussed with the regional upper GI surgical team, who recommended a further attempt at endoscopic retrieval by a senior endoscopist in the operating theatre, with the local surgical team present, due to the risk of GI perforation or injury from the retained metallic foreign body. The patient underwent a gastroscopy under general anesthesia by an endoscopist surgeon who was able to retrieve the foreign body using a snare; it was identified as a trichobezoar. Patient recovered well post-procedure, and PEG feeding was resumed. Despite the rarity of trichobezoar, it should be included in the differential diagnosis of young female patients with psychiatric disorders.

## Introduction

Trichobezoar, commonly known as a hairball, is an uncommon gastrointestinal (GI) condition that presents a notable diagnostic challenge due to its nonspecific clinical manifestations. It is characterized by the accumulation of ingested hair within the stomach, forming a compact, indigestible mass that may extend into the small intestine. When the trichobezoar extends beyond the duodenum, the condition is known as Rapunzel syndrome, a rare and potentially serious variant associated with intestinal obstruction and other complications [1].

Trichobezoars predominantly affect adolescent females, particularly those with underlying psychiatric or behavioral disorders. The condition is most commonly linked to trichotillomania, a compulsive hair-pulling disorder, and trichophagia, the habitual ingestion of pulled hair. Trichotillomania is recognized as part of the spectrum of impulse control disorders, and the associated trichophagia may occur consciously or subconsciously, often progressing unnoticed until GI symptoms arise [2]. Over time, ingested hair resists digestion and peristaltic propulsion due to its smooth, indestructible keratin surface, leading to gradual accumulation within the stomach. Patients may remain asymptomatic for extended periods, with symptoms typically emerging only when the bezoar enlarges enough to cause gastric outlet obstruction, abdominal pain, vomiting, or early satiety.

Given its subtle presentation and association with psychiatric comorbidities, trichobezoar can easily be overlooked during initial evaluation, particularly in patients who have difficulty providing reliable medical histories or cooperating with physical examination. Awareness of this condition and its behavioral associations is essential for timely diagnosis and management. We report this case to highlight the diagnostic and clinical challenges posed by trichobezoar, especially in patients with learning disabilities and psychiatric disorders, and to emphasize the importance of maintaining a broad differential diagnosis when evaluating unexplained upper GI symptoms.

## Case presentation

Patient information

A 29-year-old female with learning disabilities presented to her general practitioner with concerns regarding an obstructed percutaneous endoscopic gastrostomy (PEG) feeding tube for one week. The patient was otherwise medically stable, and her past medical history was significant only for developmental delay and dependence on PEG feeding. There was no available documentation or reliable history suggestive of behavioral disturbances, foreign body ingestion, or recent procedural complications.

Clinical findings and initial evaluation

Based on the presumed diagnosis of a dislodged or blocked PEG tube, the patient was referred for an elective upper GI endoscopy to assess the position and function of the tube. Physical examination findings were limited due to the patient’s learning difficulties and reduced ability to cooperate, which posed a diagnostic challenge; however, vital signs were normal, and full blood count and biochemistry results were unremarkable.

Endoscopic findings

**Figure 1:**
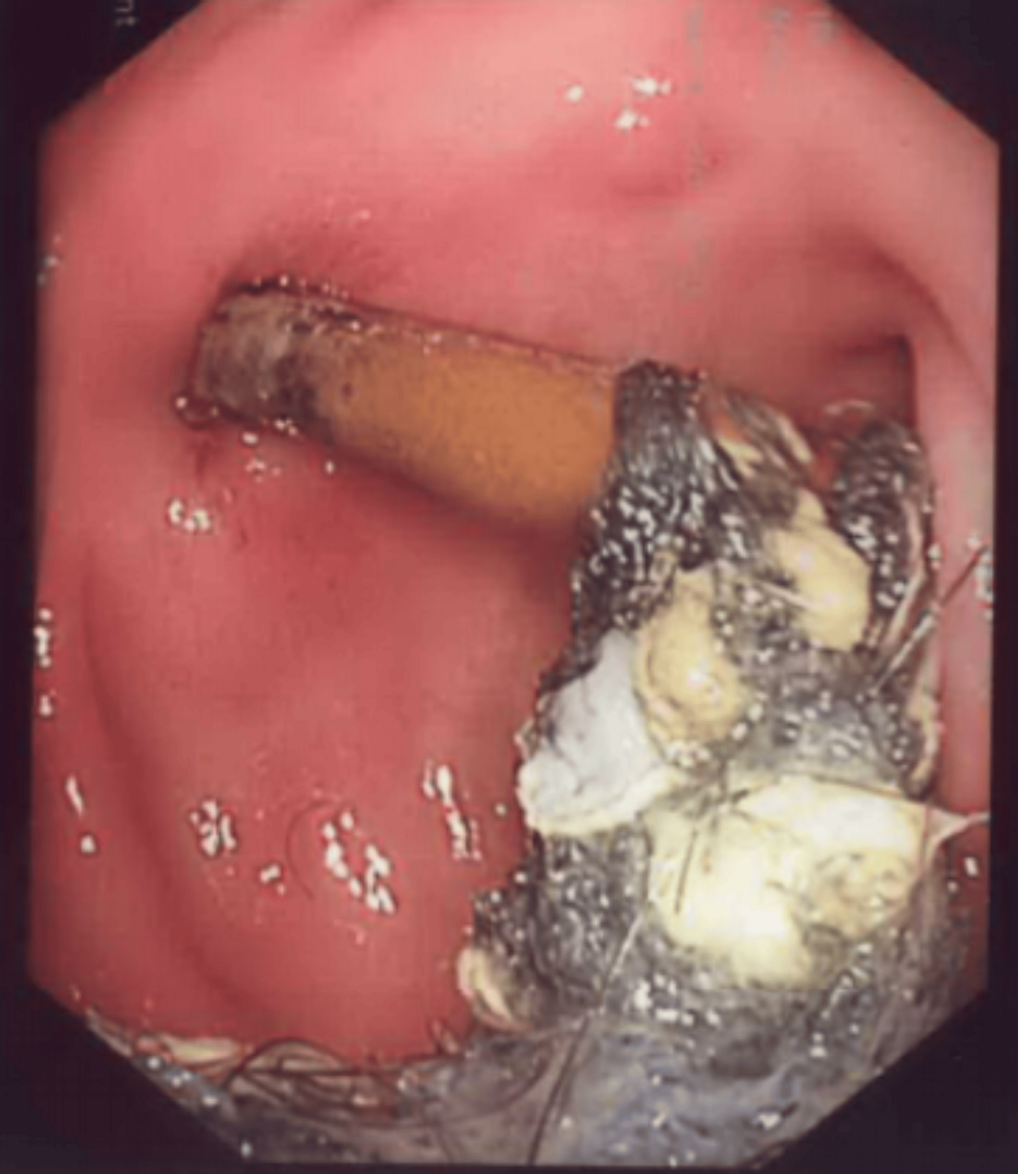
Endoscopic image of the obstructed PEG tube by trichobezoar PEG: percutaneous endoscopic gastrostomy

During gastroscopy, a foreign body resembling a metallic kitchen scrubber was visualized within the duodenal bulb (D1) and second part of the duodenum (D2), as shown in Figure 1.

**Figure 2:**
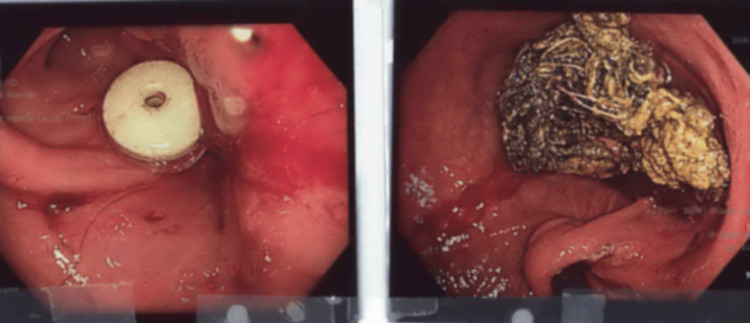
Endoscopic images of the replaced PEG tube and the residual trichobezoar PEG: percutaneous endoscopic gastrostomy

The object was found to be firmly adherent to the internal segment of the PEG tube, resulting in partial duodenal obstruction and localized ulceration of the D2 mucosa. The endoscopist successfully disengaged the PEG tube from the foreign body and replaced it with a new tube to restore enteral feeding access (Figure 2).

Procedural outcome and multidisciplinary management

**Figure 3:**
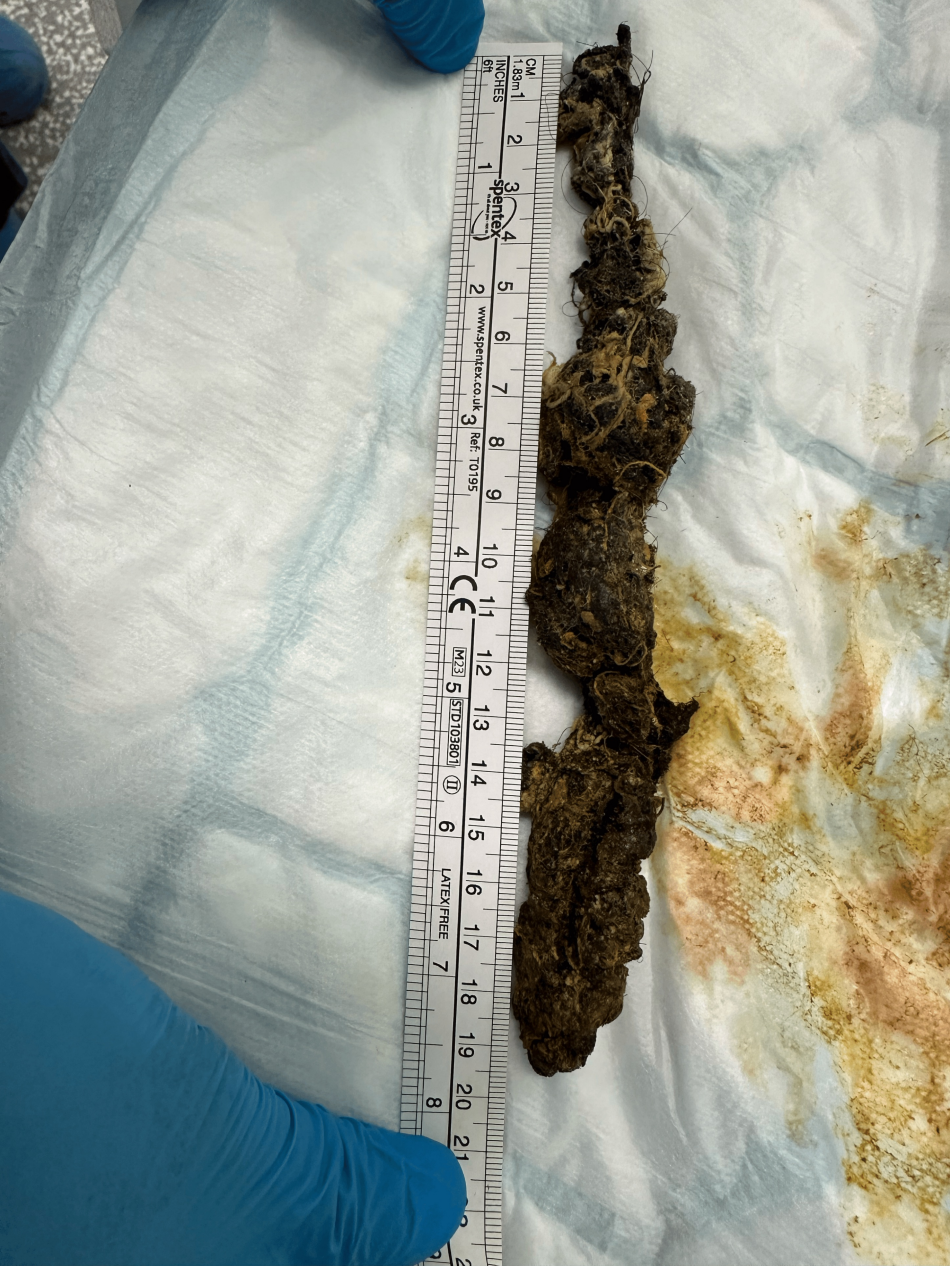
The image of trichobezoar after its removal

Despite several careful retrieval attempts, the foreign body could not be removed endoscopically during the initial procedure. A post-procedural abdominal CT scan demonstrated a presumed metallic foreign body within the stomach, measuring approximately 8 mm in maximal length and 4 mm in maximal thickness. The case was subsequently discussed with the regional upper GI surgical team, who recommended a repeat endoscopy under general anesthesia with a senior endoscopist in attendance, in collaboration with the local surgical team, to facilitate safe retrieval in a controlled theatre environment, given the potential risk of GI perforation or injury associated with the retained metallic foreign body. Fortunately, the trichobezoar was successfully removed endoscopically under general anesthesia, avoiding the need for any invasive surgical intervention (Figure 3).

The patient had an uneventful recovery after the procedure, resumed PEG feeding on the same day, and was discharged home the next day without complications. At a three-month follow-up, she continued to do well with no complaints.

## Discussion

This case highlights an important consideration for surgeons and endoscopists, as the patient initially presented with clinical features suggestive of gastric outlet obstruction. The presentation of abdominal pain secondary to trichobezoar has been documented in the literature for more than a century. Moreover, numerous reports describe trichobezoar as a cause of various abdominal symptoms encountered by both surgeons and pediatricians, indicating that the condition, although uncommon, is not rare. It should therefore be considered in the differential diagnosis of abdominal pain, particularly among patients with psychiatric disorders due to its well-established association with trichotillomania and trichophagia [3-5].

The true prevalence of trichobezoar remains unclear; however, it is frequently linked to underlying psychiatric conditions such as obsessive-compulsive disorder, body dysmorphic disorder, and depression [6-8]. A plausible explanation for the scarcity of epidemiological data is that many affected individuals are primarily referred to psychiatric services, which may limit the recognition and documentation of the physical consequences of their disorder [4]. Consequently, surgical referrals typically occur at a late stage, when patients present with pain, obstruction, or perforation [6,8,9]. Bezoars are classified into four main categories: hair (trichobezoar), milk (lactobezoar), vegetable (phytobezoar), and miscellaneous types such as those formed from fungus or sand [6]. The stomach is the most common site of occurrence, although bezoars may also be found in the duodenum, jejunum, and other segments of the intestinal tract [3,4]. In children, trichobezoars are the most frequent type, particularly among adolescent females, while in adults, phytobezoars are more prevalent [10].

The proposed pathogenesis involves the smooth surface of hair, which prevents effective propulsion by peristalsis, leading to entrapment within the gastric mucosa [10]. Normally, the stomach is capable of clearing even large foreign bodies in up to 80-90% of cases, suggesting that bezoar formation likely requires two contributing factors: an alteration in gastric anatomy or physiology and the continuous ingestion of indigestible material [7,11]. The clinical presentation of trichobezoar varies from acute abdomen to GI obstruction, with common symptoms including abdominal pain, nausea, vomiting, hematemesis, early satiety, and the presence of a palpable mass, depending on the level of obstruction [6,8,9].

Our patient's initial presentation was related to an obstructed PEG feeding tube; moreover, the patient's mother reported intermittent upper abdominal pain and episodes of vomiting. Diagnostic evaluation can be aided by imaging modalities. Ultrasonography may identify epigastric masses, while CT is more accurate in delineating the characteristic features of a bezoar. Given our patient's young age and limited history, endoscopy was performed initially to assess the PEG tube and evaluate for gastritis. The procedure revealed a foreign body surrounding the PEG tube, partially obstructing the pylorus and explaining the recurrent vomiting. Given the unclear history and uncertain nature of the gastric mass, a CT scan was subsequently performed for further clarification and to guide management planning, including potential laparoscopic or open removal. Fortunately, the bezoar was successfully removed endoscopically under general anesthesia, avoiding the need for invasive surgical intervention.

This report underscores the diagnostic challenges encountered in patients with psychiatric disorders or learning disabilities, in whom obtaining a reliable history and ensuring cooperation during examination are often difficult. Such factors can delay recognition and appropriate management of rare but significant conditions like trichobezoar.

## Conclusions

Key learning points from this report include the importance of routinely inquiring about hair ingestion habits and their frequency during history-taking in children and individuals with behavioral or psychiatric issues. In addition, clinicians should examine common sites of hair pulling for evidence of alopecia. When a trichobezoar is suspected in the differential diagnosis of acute abdomen or gastric outlet obstruction, it should be discussed with the radiology team to ensure focused assessment for potential diagnostic signs on imaging. Ultimately, this report highlights the need to broaden the differential diagnosis in cases of unexplained abdominal pain or obstruction and to remain vigilant for unusual presentations, particularly in patients with psychiatric or cognitive challenges, where history-taking and examination may be limited.
